# Energy intake and the circadian rhythm of core body temperature in sheep

**DOI:** 10.1002/phy2.118

**Published:** 2013-10-23

**Authors:** Shane K Maloney, Leith C R Meyer, D Blache, A Fuller

**Affiliations:** 1 Anatomy Physiology and Human Biology, The University of Western AustraliaPerth, Australia; 2 Brain Function Research Group, School of Physiology, University of the WitwatersrandJohannesburg, South Africa; 3 Department of Paraclinical Science, University of PretoriaPretoria, South Africa; 4 Institute of Agriculture, The University of Western AustraliaPerth, Australia

**Keywords:** Energy balance, heterothermy, homeothermy, nutrition, thermoregulation

## Abstract

We tested the hypothesis that different levels of energy intake would alter the circadian rhythm of core body temperature (T_c_) in ovariectomized sheep. We measured arterial blood temperature every 5 min while ten sheep were offered a maintenance diet, 70% of maintenance requirements, or 150% of maintenance requirements, for 12 days, and later fasted for 2 days. The rhythmicity of T_c_ was analyzed for its dominant period and then a least-squares cosine wave was fitted to the data that generated a mesor, amplitude, and acrophase for the rhythm. When energy intake was less than maintenance requirements we observed a significant decrease in the mesor and minimum, and a significant increase in the amplitude and goodness of fit, of the body temperature rhythm. Fasting also resulted in a decrease in the maximum of the body temperature rhythm. Feeding the sheep to excess did not affect the mesor or maximum of the rhythm, but did result in a decrease in the goodness of fit of the rhythm in those sheep that consumed more energy than when they were on the maintenance diet, indicating that circadian rhythmicity was decreased when energy intake increased. Our data indicate that modulation of the circadian rhythm of body temperature, characterized by inactive-phase hypothermia, occurs when energy intake is reduced. The response may be an adaptation to energy imbalance in large mammals.

## Introduction

By definition, homeothermic animals maintain a core body temperature (T_c_) that oscillates in a narrow range, even when ambient temperature oscillates widely (IUPS Thermal Commission 2001). The T_c_ exhibits an endogenous daily cycle, or circadian rhythm, usually with higher T_c_ during the active phase of an animal's daily cycle and lower T_c_ during the inactive phase (Aschoff 1982; Refinetti and Menaker 1992). When ambient temperature decreases, the maintenance of homeothermy requires that an animal balances increased heat loss with elevated heat production, an energetically expensive process. On occasion, some small mammals and birds abandon homeothermy and enter a regulated hypothermia, known as torpor or hibernation, reducing the metabolic cost of thermoregulation and accruing energy savings because metabolism decreases via the *Q*_10_ effect (Geiser 2004).

The entry into torpor or hibernation occurs generally at a time when energy balance is compromised, by either increased energy costs (low ambient temperature) or reduced energy supply. The capacity of small birds and mammals to enter a regulated hypothermia in response to starvation is well characterized, and is considered an important adaptation that reduces energy expenditure on thermoregulation when energy supply is attenuated (Lyman et al. 1982; Hohtola 2012). With the notable exception of the black bear (Toien et al. 2011), most hibernators are relatively small with a median body mass of 85 g (Turbill et al. 2011). But there are examples of lower than normal T_c_ (which by definition is hypothermia) in large mammals and birds in response to starvation. This hypothermia has been observed to develop gradually with food deprivation in goats, sheep, dogs, rhesus monkeys, and juvenile king penguins (Piccione et al. 2002, 2003, 2005; Eichhorn et al. 2011). We (Fuller et al. 2005; Maloney et al. 2011) and others (Arnold et al. 2004, 2006; Grigg et al. 2009) have also observed changes in the T_c_ rhythm, especially an increase in the amplitude resulting from hypothermia during the inactive phase of the daily rhythm, in several large free-ranging mammals when they have access to food, meaning that the hypothermia we have observed cannot be a response to starvation. The hypothermia may be a response to a reduction, rather than a cessation, in feed quality or intake. It is known that long-term calorie restriction results in a decrease in subcutaneous temperature in rhesus monkeys (Lane et al. 1996), and that restricted feeding leads to a lower rectal temperature in the morning in Sudanese goats (Ahmed and El Kheir 2004) and Shetland ponies (Brinkmann et al. 2012), and a lower vaginal temperature in Suffolk ewes (Sudarman and Ito 2000). The observation in some species that the amplitude of the daily T_c_ rhythm is larger in winter could also be a response to season (day length), or it may perhaps be a response to increased energy expenditure during rutting, pregnancy, or lactation. Feed intake could also affect the body temperature via the heat increment of feeding, hypothesized to have played a role in the hypothermia observed in red deer because the protein content of the feed was lower, when hypothermia was observed, in the winter (Arnold et al. 2004). Hypothermia in camels could also be an adaptation that preempts the metabolic heat load that occurs during male–male fights during the seasonal rut (Grigg et al. 2009), whereas Maloney et al. (2011) hypothesized that energy intake *per se* played a role in summer hypothermia in gray kangaroos because the summer corresponded to the dry season, resulting in decreased feed quality and quantity where the study was conducted.

Understanding the mechanism of hypothermia in large mammals is important in interpreting the ecological significance of the altered physiology. For example, if inactive-phase hypothermia in winter was the result of an inability to thermoregulate, then it would have very different ecological consequences than if the hypothermia was regulated. If the response was due to a lack of thermoregulatory capacity in the cold, then presumably the animal would be operating at maximal cold-induced metabolism, but that level of heat production would be insufficient to balance heat loss, resulting in hypothermia. On the other hand, if the hypothermia was regulated, as it is in torpor, then metabolism would be minimal during the nadir of the T_c_ rhythm, and lower than normal because of the *Q*_10_ effect associated with a lower than normal T_c_. The implications for the energy requirements of animals are very different depending on the mechanism.

We here test the hypothesis that the level of energy intake can affect the circadian pattern of thermoregulation in a relatively large-sized mammal. We use sheep as the animal model and expose them to a constant ambient temperature well within their thermoneutral zone and so prevent the possibility that any response is due to an inability to thermoregulate. We then alter the food offered to the sheep while measuring their T_c_ and analyze the resulting rhythms. We test the effect of a decrease, and then a cessation, in energy intake to determine whether hypothermia results, and also test the effect of an increase in energy intake to determine whether the heat increment of feeding alters the T_c_ rhythm in thermoneutral conditions.

## Material and Methods

### Ethics

The University of Western Australia animal ethics committee approved all the experimental work (AEC 100/594). During experiments the sheep were housed in individual pens (∼1.6 × 0.8 m) at the large animal facility at the University of Western Australia. The facility is air conditioned and the sheep were exposed to a constant 22–23°C and a light:dark cycle of 12:12 h with lights on at 07:30. The sheep were not shorn prior to the experimentation and were carrying about 5 cm of fleece. For sheep with that level of fleece cover, 22°C is well within the thermoneutral zone (Blaxter et al. 1958, 1959). Relative humidity in the rooms was not controlled but averaged around 50%, meaning that the sheep were exposed to a temperature–humidity index of about 68.

### Animals

Twelve Merino ewes (*Ovis aries*) were used in the study, with data from ten used for analysis. The ewes were ovariectomized at least 2 weeks prior to experimentation to remove the cyclic influences of ovarian steroids on T_c_. At the commencement of experimentation, the body mass of the 10 sheep used for analysis was 49.0 ± 1.1 kg.

### Surgery

Anesthesia was induced in the sheep with thiopentone sodium (15 mg kg^−1^, Thiobarb; Jurox, Rutherford, Australia) injected intravenously, and maintained with isoflurane (1–2%, Attane; Pharm Tech, West Pymble, Australia) in oxygen administered in the inspired gas via an endotracheal tube. Respiratory rate, peripheral hemoglobin oxygen saturation, heart rate, and rectal temperature were monitored throughout the surgical procedure, which lasted ∼30 min.

We measured T_c_ using a thermistor inside a blind-ended and thin-walled polytetrafluoroethylene (PTFE) tube (OD 1.35 mm, ID 0.97 mm; Straight Aortic Flush 4F Catheter, Cordis, The Netherlands) placed into the left carotid artery. The thermistor was connected to an insulated extension lead attached to a data logger (see Temperature measurements section) implanted in the neck. Using sterile surgical conditions, we implanted the thermistor probe and logger. The loggers and probe were dry sterilized in formaldehyde vapor before implantation. An incision was made along the neck after local injection of lignocaine with adrenaline (1–2 mL of 20 mg mL^−1^ lignocaine, 0.018 mg mL^−1^ adrenaline; Troy laboratories, Smithfield, Australia) to isolate the left carotid artery. The thermistor was positioned in the artery midway along the length of the neck, and advanced 100 mm, toward the heart. The base of the thermistor probe was secured by a purse-string suture in the vessel wall, while the remainder of the probe lay free in the arterial lumen. Postoperatively the sheep received a long acting antibiotic (Benacillin; Troy Laboratories, Smithfield, Australia) and anti-inflammatory (3.5 mg kg^−1^ of carprofen; Rimadyl; Pfizer, West Ryde, Australia).

At the end of experimentation, the sheep were killed with an overdose of barbiturate anesthetic and the thermistors and data loggers were retrieved, recalibrated, and the data downloaded.

### Temperature measurements

The miniature thermometric data loggers (StowAway XTI; Onset Computer, Pocasset, Massachusetts) that were used to measure carotid blood temperature had outside dimensions of ∼50 × 45 × 20 mm and a mass of ∼40 g when covered in inert wax (Sasol, Johannesburg, South Africa). These loggers had a resolution of 0.04°C and measurement range from +34°C to +46°C. The temperature sensors used to measure carotid blood temperatures were constructed from ruggedized glass-coated bead thermistors with insulated extension leads (bead diameter 0.3 mm; AB0E3-BR11KA103N, Thermometrics, Edison, NJ). All temperature sensors and loggers were calibrated against a certified mercury in glass thermometer (National Association of Testing Authorities, Rhodes, NSW, Australia) between 30°C and 42°C and programmed to record temperature every 5 min. After calibration, the loggers and their sensors measured blood temperature to an accuracy of better than 0.05°C.

### Experiment 1: The effect of feeding in excess to, or less than, maintenance requirements

Prior to experimentation, the 12 sheep were brought into the Large Animal Facility and acclimatized to the experimental procedures, including handling for the determination of body mass and feed access being restricted to 7 h per day, for 2 weeks. Surgery for the implantation of recording devices was performed during the third week and then at least 10 days recovery was allowed before experimentation began. Throughout the acclimatization, the sheep were fed at 09:00 and the residual feed was removed (and weighed) at 16:00. The sheep were fed a maintenance diet of lucerne chaff and lupin seed, provided according to an individual's maintenance requirements. Maintenance requirements were calculated as predicted by the daily energy requirement (MJ) based on body mass and predicted growth rate (50 g day^−1^). The maintenance diet (referred to as “Maintenance” diet) for a 49 kg animal was 800 g of lucerne chaff and 80 g of lupin seed, providing 7.25 MJ day^−1^ of metabolizable energy, which is 0.14 MJ kg^−1^ day^−1^, with ∼185 g day^−1^ of crude protein. This level of metabolizable energy intake is equivalent to the “medium” diet used previously by several groups to study thermoregulation in sheep (Blaxter et al. 1958, 1959; Graham et al. 1959; Sudarman and Ito 2000).

To examine the effects of high and low energy intake, six sheep were randomly chosen to receive 1.5× the maintenance energy requirements, whereas the other six sheep received 0.7× the maintenance energy requirements. These diets were chosen based on the level of intake that has been used previously to study the effects of under- and overnutrition in sheep thermoregulation (Blaxter et al. 1958, 1959; Graham et al. 1959; Sudarman and Ito 2000), and are referred to as “1.5” and “0.7” diets, respectively. The sheep on the 1.5 diet were offered 1050 g day^−1^ of lucerne chaff with 200 g day^−1^ of lupin grain, providing 10.5 MJ day^−1^, which is 0.21 MJ kg^−1^ day^−1^, with ∼275 g day^−1^ of crude protein, whereas those on the 0.7 diet were offered 550 g day^−1^ of lucerne chaff and 60 g day^−1^ of lupin grain providing 4.9 MJ day^−1^, which is 0.10 MJ kg^−1^ day^−1^, with ∼130 g day^−1^ of crude protein. The sheep were provided with these diets for 12 days, after which they were returned to maintenance feeding for 12 days. The 1.5 and 0.7 diets were then reinitiated using a crossover design, so that each sheep received the diet manipulation other than that it had received for the first 12 days. Thus, each animal received each diet during the experiment. Food intake was measured daily and body mass was measured on the days that dietary manipulation began and ended. Food intake was calculated each day by measuring the residual food at 16:00, and intake was then converted into the energy consumed (MJ). The change in body mass on each diet was calculated by subtracting the body mass on day 12 of each diet from the body mass measured on day 0 of each diet.

### Experiment 2: The affect of fasting

We examined the effects of fasting by removing feed, on separate occasions, for 24 and 48 h. The first day of fasting was 12 days after the end of experiment 1, and during this baseline period the maintenance diet of lucerne chaff and lupin seed was offered at 09:00 as in the previous experiments. On day 1 of the experiment all the sheep were fasted, that is, food was not offered at the scheduled time. On the following day half of the sheep were fed, whereas the others remained fasting for a further 24 h. On the following days all the sheep were fed the maintenance diet. The design was repeated 4 days later in a crossover design so that all the sheep underwent both 24 and 48 h fasting on separate occasions.

### Data and statistical analyses

The thermistor that measured carotid temperature in one of the sheep malfunctioned within 10 days after surgery. Another sheep became ill and had to be removed from the study. Therefore, we were able to analyze changes in the T_c_ rhythm for 10 sheep. The dominant period of the data series was determined using periodogram analysis (Sokolove and Bushell 1978). A 24 h dominant period was found and was then assumed for cosinor analysis (Nelson et al. 1979), which was used to characterize the parameters of the rhythm (mesor, amplitude, and acrophase) of T_c_ for each treatment.

For each treatment in experiment 1 (0.7 maintenance, and 1.5 diets) the characteristics of the cosinor were calculated as an average for the last 7 days, from midday on day 5 to midday on day 12, for each animal on each diet. For experiment 2, the characteristics were calculated from midday to midday for each day for each animal. The data from each animal when they were fasted for 24 h was used to calculate the “fasting day 1” (“F1”) averages, whereas the data from day 2 of the 48 h fast was used to calculate the “fasting day 2” (“F2”) averages. We tested whether the order of experimentation had any effect on the measured parameters; in experiment 1 whether feeding the 1.5 or 0.7 diet first, and in experiment 2 whether 24 or 48 h of fasting first, had an effect. We initially used a two-way repeated measures analysis of variance (ANOVA, main effects of treatment and order of experiment) to compare the different treatments of experiment 1 and the two conditions of fasting of experiment 2 for the mean level (mesor), amplitude, minimum and maximum level, and acrophase of the T_c_ rhythm. We also calculated a goodness of fit of the cosinor by calculating the sums of squares of the residuals of the fitted versus the original data (actual T_c_ minus fitted T_c_) for each animal as:


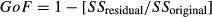


Order of treatment was not significant for any variable and so we removed that effect and performed a one-way repeated measures ANOVA across all the treatments (1.5, maintenance, 0.7, F1, and F2). When significant differences between treatments were indicated by the ANOVA, these differences were explored with a Student–Newman–Keuls (SNK) post hoc test. Data in figures and tables are presented as mean ± SD.

## Results

On the maintenance diet the sheep consumed 0.145 ± 0.008 MJ kg^−1^ day^−1^ (Table 1). On the 0.7 diet they consumed 62% of the energy consumed on the maintenance diet, and on the 1.5 diet they consumed 127% of the energy consumed on the maintenance diet. When they were on the maintenance diet for the middle 12 days of experiment 1, the sheep gained 1.4 kg (Table 1). On the 0.7 diet the sheep lost 2.5 kg in 12 days, whereas on the 1.5 diet they gained 1.6 kg. When they were placed on the maintenance diet for the middle 12 days of experiment 1, the sheep that had been on the 0.7 diet for the first 12 days of the experiment gained significantly more body mass than the sheep that had been on the 1.5 diet for the first 12 days of the experiment (2.2 vs. 0.2 kg; *P* = 0.004). Because the two sheep that were removed from analysis had been on the 1.5 diet for the first 12 days, the statistics for the maintenance period were biased toward sheep that had been on the 0.7 diet for the first 12 days. Perhaps because of this bias, the mass gain of the sheep on the 1.5 diet was not different from the mass gain on the maintenance diet, but the mass gain on those two diets was significantly higher than when the sheep were on the 0.7 diet (Table 1).

**Table 1:** Energy intake and change in body mass of sheep exposed to thermoneutral ambient temperature that were fed for 12 days a diet that supplied either maintenance energy requirements, or 0.7 or 1.5× maintenance energy requirements

	0.7 diet	Maintenance diet	1.5 diet
Energy intake (MJ day^−1^)	4.4 ± 0.85^a^	7.0 ± 0.40^b^	9.1 ± 1.12^c^
Energy intake (MJ kg^−1^ day^−1^)	0.089 ± 0.017^a^	0.145 ± 0.008^b^	0.184 ± 0.020^c^
Energy intake (% maintenance)	62	100	127
Mass change (kg)	−2.5 ± 1.1^a^	1.4 ± 1.2^b^	1.6 ± 1.0^b^
Mass change (%)	−4.9 ± 2.2^a^	3.0 ± 2.7^b^	3.3 ± 2.0^b^

Data are presented as mean ± SD. Means with different superscripts across a row differ significantly (*P* < 0.05).

**Figure 1:**
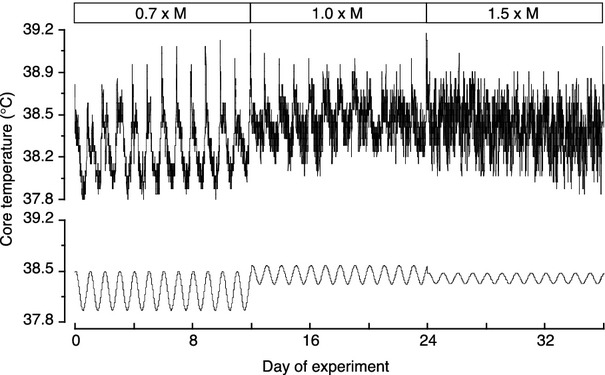
Body temperature rhythm during experiment 1. Original record of 5-min recordings of body temperature (top panel), and the associated fitted cosinor (bottom panel) from a representative sheep under experimental conditions in which the food offered was changed from 0.7–1.0 to 1.5× the maintenance requirements.

**Figure 2:**
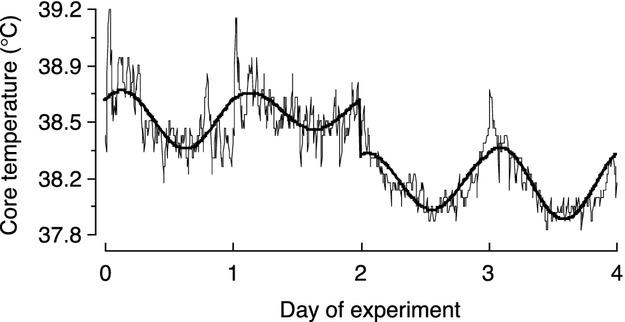
Body temperature rhythm during experiment 2. Original record of 5-min recordings of body temperature (thin line), and the associated fitted cosinor (thicker line), from a representative sheep under experimental conditions in which food was withdrawn after day 2. The sheep had been offered a maintenance diet on the first 2 days.

Throughout all experimental procedures, the sheep displayed a robust rhythmicity in T_c_ within each 24-h period. The pattern of the T_c_ rhythm for a single representative animal, throughout the successive 12-day periods in which this animal was provided with 0.7, maintenance, and then 1.5 diets, is shown in Figure 1 (upper panel). Although there was large variability in the original 5-min recordings of T_c_, the cosinor analysis highlighted the general changes in the T_c_ rhythm across the different feed intake regimes with an apparent inverse relationship between energy intake and the amplitude of the rhythm (Fig. 1, lower panel). The forced cessation of feed intake during experiment 2 resulted in further changes in the T_c_ rhythm, with a lowering of the rhythm of T_c_ on the first day of starvation (day 2 on Fig. 2), that was amplified on the second day of starvation (day 3 on Fig. 2).

**Figure 3:**
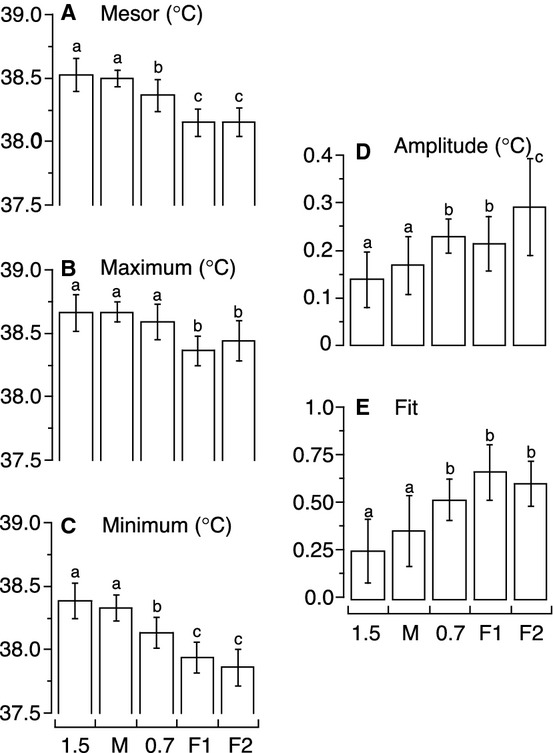
Characteristics of body temperature. (A) Mesor, (B) maximum, (C) minimum, (D) amplitude, and (E) goodness of fit of the daily rhythm of body temperature (mean ± SD, *n* = 10) over the five experimental conditions, namely, 1.5× maintenance (1.5), maintenance (M), or 0.7 of maintenance (0.7), day 1 of fasting (F1) and day 2 of fasting (F2). Means that proved to differ by SNK testing (*P* < 0.05) have different superscripts.

To assess these changes in T_c_ patterns, we characterized the T_c_ rhythm under the different feed intake regimes (Fig. 3). The mesor of the T_c_ rhythm was reduced under conditions in which feed intake was below maintenance (Fig. 3A, *F*_4,36_ = 43.5, *P* < 10^−6^), both during the 12-day period when the sheep were on the 0.7 diet (0.7 vs. maintenance; SNK *P* < 0.001) and to an even greater extent when feed was removed entirely (both F1 vs. maintenance and F2 vs. maintenance; SNK *P* < 0.001). The maximum of the rhythm was affected by energy intake (Fig. 3B, *F*_4,36_ = 21.4, *P* < 10^−6^) only during fasting (both F1 vs. maintenance and F2 vs. maintenance; SNK *P* < 0.001). The reduction in the mesor of the T_c_ rhythm was associated with a lowering of the minimum of the T_c_ rhythm (Fig. 3C, *F*_4,36_ = 44, *P* < 10^−6^) when the sheep were on the 0.7 diet (maintenance vs. 0.7; SNK *P* < 0.001) and further reduced on both days of fasting (both F1 vs. maintenance and F2 vs. maintenance; SNK *P* < 0.0001). The respective changes in maximum and minimum temperatures resulted in a larger amplitude of the T_c_ rhythm when feed intake was below maintenance requirements (Fig. 3D, *F*_4,36_ = 11.8, *P* = 3 × 10^−6^). The higher amplitude of the T_c_ rhythm was particularly obvious after the sheep had been deprived of food for 2 days (F2 vs. maintenance; SNK *P* < 0.0001).

The timing of the rhythm was not affected by diet (*F*_4,36_ = 2.17, *P* = 0.09), with the acrophase of the rhythm on all diets occurring at 12:47 ± 1.6 h. Thus, the acrophase of the T_c_ rhythm occurred during the day when the lights were on and the sheep had access to food. The nadir occurred when the lights were off at 00:47, and when the sheep had no access to food. The fit of the sinusoid model improved under all conditions in which feed intake was below maintenance requirements (Fig. 3E, *F*_4,36_ = 14.7, *P* = 3 × 10^−6^). There was a tendency for the fit of the sinusoid model to decrease on the 1.5 diet (maintenance vs. 1.5; SNK *P* = 0.10). On the 1.5 diet one of the sheep consumed only 7.6 MJ day^−1^, which was close to the level provided during the maintenance diet. If that single animal is removed from the analysis, then the goodness of fit on the 1.5 diet was significantly lower than on the maintenance diet (*t*_8_ = 3.2, *P* = 0.01).

## Discussion

If the normal T_c_ rhythm is defined as that predominating when the sheep were fed at maintenance requirements and exposed to ambient temperature within the thermoneutral zone, then offering the sheep 70% of the energy they received on the maintenance diet led to nocturnal hypothermia with no change in the diurnal T_c_. In this respect our results show that a decrease in energy intake leads to the same phenomenon as does fasting, where the effects were predominantly on the T_c_ during the inactive phase of the daily rhythm, meaning that there is circadian modulation of hypothermia in sheep (Piccione et al. 2002). Furthermore, the goodness of fit of the cosinor model increased significantly when energy intake was attenuated, indicating that the pattern became more cosine wave like associated with an increase in the amplitude of the T_c_ rhythm. In closely clipped sheep, the level of resting heat production was lower when the sheep were fed 55% of maintenance requirements, and higher when they were fed 140% of maintenance requirements, but those changes in heat production were offset by changes in heat loss and there was no effect on rectal temperature (Blaxter et al. 1958; Graham et al. 1959). The lower critical temperature (LCT; the ambient temperature below which a homeotherm increases metabolic heat production to balance increased heat loss and maintain body temperature) of the sheep did change with feeding level (being higher when the sheep were intake restricted), but when the sheep had a covering of fleece >2.5 cm, the LCT was below 10°C (Blaxter et al. 1959). It therefore appears that sheep with even a moderate covering of fleece have the capacity to maintain T_c_ at a wide range of ambient temperatures, despite changes in feed intake of between 55 and 140% of maintenance levels, at least during the daytime, when these previous studies were conducted. What we observe here is an apparent change in the regulatory system of the sheep in a way that sees them not defend the normal body temperature during the nocturnal phase when feed intake is reduced. The complete cessation of intake by 2 days of fasting amplified the changes observed during attenuated intake on the 0.7 diet, with the addition that the maximum of the rhythm decreased. On the other hand, when the sheep consumed more than the maintenance requirements, the fit of the sinusoid model to the data decreased, whereas the mean and amplitude of the daily T_c_ rhythm did not change.

The T_c_ rhythm was altered by both an increase and a decrease in energy intake relative to the maintenance diet. What the mechanism of these changes is remains to be elucidated. Arnold et al. (2004) proffered that an alteration in the heat increment of feeding explained seasonal differences in peripheral temperatures in red deer. The heat increment of feeding is the heat produced as a by-product of the inefficiency of digestion and absorption processes (Blaxter 1989). This mechanism is unlikely to explain our results in sheep because feeding, and therefore any alteration in the heat increment of feeding, occurred during the day, which is when the T_c_ was highest, and the maximum T_c_ was the same on the 1.5, maintenance, and 0.7 diets. The major changes in T_c_ that we observed occurred during the night when the sheep had no access to food. The only treatment that affected the diurnal temperature was fasting. It remains possible that the decrease in the daily maximum of the T_c_ rhythm during starvation resulted from the removal of the heat increment of feeding.

It is unlikely that the reduction in the minimum T_c_ on the 0.7 diet was due to an inability of the sheep to produce sufficient metabolic heat to maintain T_c_, as even complete fasting for 24 h did not affect the summit metabolism in sheep, and summit metabolism was reached only when sheep were exposed to wind at 1°C and wetted (Bennett 1972), whereas our sheep were exposed to 22°C, which would have required a heat production well below summit metabolism. To maintain metabolism at the same level during the 0.7 diet as during the maintenance diet would have required the use of body energy stores. The difference in energy intake between the maintenance and 0.7 diet was 2.8 MJ day^−1^ (7.25–4.45 MJ day^−1^). That would require about 75 g of fat per day, or about 1 kg of fat over the 12 days. As the sheep lost an average of 2.5 kg during the 0.7 diet period, such fat metabolism may have occurred. However, part of the decline in mass could simply be due to changes in rumen fill, and the water associated with rumen fill, rather than a change in dry body mass. Another possibility is that metabolism decreased. Feed restriction is known to lead to a reduction in resting metabolism in several artiodactyl species, the Bedouin goat (Choshniak et al. 1995), the Arabian oryx (Ostrowski et al. 2006), and Suffolk ewes (Sudarman and Ito 2000). Such a metabolic response may have been responsible for the “compensatory growth” (sometimes called “catch-up growth”) during the maintenance feeding period in the sheep that had immediately previously been on the 0.7 diet. Those sheep gained 2.2 kg in 12 days (183 g day^−1^) on a diet that was designed to permit growth at 50 g day^−1^, suggesting that maintenance energy requirements coming of the 0.7 diet were lower than when the animals were fed at maintenance. At the same maintenance energy intake, the sheep that had been on the 1.5 diet for the preceding 12 days gained only 0.2 kg (16 g day^−1^), suggesting to us that the sheep coming off the 0.7 diet carried a lower metabolic rate into the maintenance feeding period than did the sheep that had been on the 1.5 diet. The phenomenon of compensatory growth in response to a period of feed restriction is well known, and usually involves an increase in food consumption during the compensatory phase (Wilson and Osbourn 1960). Our sheep could not increase feed consumption because we offered them only maintenance energy requirements, yet they exhibited accelerated mass gain. Alternatively, our sheep could have carried over an increase in metabolic efficiency (mass gain per unit of energy consumed) developed during the restriction period, a phenomenon reported in sheep during compensatory growth in the absence of increased energy intake (Ryan et al. 1993).

In many of the sheep (including the one illustrated in Fig. 1), the 1.5 diet led to an apparent loss of fine control of the T_c_ rhythm. The rhythm appeared to oscillate within the same maximum and minimum as on the maintenance diet, but became more noisy. For that assertion to have been statistically supported, the goodness of fit should have decreased. In fact there was a trend toward an increase in noise on the 1.5 diet (maintenance vs. 1.5; SNK *P* = 0.10), and by removing one animal from the analysis that ate only as much as it did on the maintenance diet during its time on the 1.5 diet that trend changed to a significant decrease in the goodness of fit on the 1.5 diet. A loss of rhythm coincident on increased food intake may have important implications because it appears that the T_c_ changes associated with the daily rhythm can act as a zeitgeber for clocks in peripheral tissues, whereas the “master clock” in the suprachiasmatic nucleus operates independently of temperature (Buhr et al. 2010). If an increase in energy intake disrupts circadian signaling to peripheral tissues via the circadian rhythm of T_c_, then it seems likely that metabolic systems will be disturbed.

Evidence emerging from the study of neuropeptides in the brain suggests a strong interaction between appetite control and thermoregulation. Neuropeptide-Y (NPY) is best known for its potent orexigenic effect, stimulating food intake behavior in mammals, including sheep (Miner et al. 1989). The injection of NPY into the hypothalamus of rodents not only stimulates appetite but also leads to a reduction in metabolic rate (Walker and Romsos 1993) and a decrease in core temperature (Bouali et al. 1995). The same procedure in Siberian hamsters induces torpor (Paul et al. 2005). It is apparent that the same neuropeptide, released in response to food deprivation or starvation, stimulates eating behavior as well as reducing energy expenditure on thermoregulation. If NPY has similar effects in large mammals, it would suggest that in large animals the rhythm of T_c_ is modified by energy intake, and that the altered temperature pattern leads to energy savings. We concur with Arnold et al. (2004) who concluded that the reduction in energy expenditure and the abandonment of the defense of high T_c_ in response to restricted energy intake may be a response that is not restricted to hibernators and daily heterotherms, but may be a common physiological response of terrestrial mammals.

Combined with the recent finding that the predominant driver of heterothermy in the desert dwelling Arabian oryx was not environmental heat load, but was water availability (Hetem et al. 2010), we propose that the characteristics of the T_c_ rhythm provide not only a measure of thermal strain but also manifest when either osmotic or energy strain is experienced by an animal. The implication is that mammals that are exposed to ideal conditions should have a smaller amplitude of the T_c_ rhythm than animals that are exposed to any thermal, osmotic, or energy stress. Whether other stressors similarly affect the amplitude of the T_c_ rhythm remains to be elucidated.
